# Sexual behaviours of men who inject drugs in Northeast India

**DOI:** 10.1186/s12954-015-0038-1

**Published:** 2015-03-04

**Authors:** Michelle Kermode, Greg Armstrong, Gajendra Kumar Medhi, Chumben Humtsoe, Biangtung Langkham, Jagadish Mahanta

**Affiliations:** Nossal Institute for Global Health, University of Melbourne, Level 4, 161 Barry St, Carlton, Victoria 3010 Australia; Regional Medical Research Centre, Indian Council for Medical Research, Post Box No.105, Dibrugarh, 786001 Assam India; Emmanuel Hospital Association, 808/92 Deepali Building, Nehru Place, New Delhi 11019 India

**Keywords:** HIV, People who inject drugs, India, Sexual partners

## Abstract

**Background:**

Promoting safer sex behaviours among people who inject drugs is important as drug-using populations with high HIV prevalence can contribute to transition from a concentrated to a generalised epidemic. This study describes the sexual behaviours of men who inject drugs in two Northeast Indian states (Manipur and Nagaland) where HIV prevalence is high, with a focus on the HIV risks for their regular female sexual partners.

**Methods:**

Data were obtained from two cross-sectional surveys combined (*N* = 3,362)—both conducted in 2009 using respondent-driven sampling to recruit men who injected drugs. Both surveys asked about demographics, drug use, sexual and injecting risk behaviours, and interventions. One survey tested participants for HIV and syphilis. Statistical analyses included logistic regression modelling to predict inconsistent condom use with regular sexual partners.

**Results:**

Two thirds of participants (68.2%) had a regular female sexual partner. Of these, 78.4% had sex with their regular partner in the last month, on average five times. Only 10.7% reported consistent condom use with regular partners. Unsafe injecting was common among men with regular partners, and 40.2% had more than one sexual partner in the last year. Half of those with regular partners (51.0%) had never had an HIV test, and 14.3% of those tested were HIV positive. After controlling for confounding, inconsistent condom use with regular partners was associated with being illiterate, married, sharing needle and syringe with others, never having had an HIV test and not receiving condoms from an NGO.

**Conclusion:**

The findings from this study among men who inject drugs in Manipur and Nagaland highlight the risk of HIV infection for their regular female sexual partners. Promoting better uptake of HIV testing among men who inject drugs will potentially benefit both them and their regular partners. While effectively reaching regular partners is challenging, a number of strategies for improving their situation in relation to HIV prevention are available.

## Background

There are an estimated 16 million people who inject drugs (PWID) in the world, of which three million are thought to be HIV positive [1]. While the injecting behaviours of PWID are relatively well documented, much less is known about their sexual behaviours, and it is likely that sexual behaviours among PWID vary depending on the context. Most HIV prevention programs targeting PWID focus primarily on the promotion of safe injecting practices and the distribution of injecting equipment and are less vigilant about preventing sexual transmission of HIV [2,3]. The prevention of sexual transmission from PWID to their regular partners is important as PWID populations with high HIV prevalence can contribute to the transition from a concentrated HIV epidemic mainly affecting particular population sub-groups (such as PWID, men who have sex with men, and female sex workers) to a more generalised heterosexual epidemic as has possibly happened in Argentina, Brazil, China, Indonesia, Netherlands and Ukraine [4].

The majority of PWID in India are male and overall 7% are HIV positive, but this varies substantially depending on location. For example, prevalence in the state of Punjab is 21%, while in other states, such as Karnataka, it is negligible [5]. Many men who inject drugs in India have regular female sexual partners (henceforth referred to as regular partners), most of whom are at high risk of HIV infection due to unprotected sex with their regular (injecting) partner/husband [6,7]. Studies in Chennai [8-10], Manipur [11-13], Delhi [11,14], and nationally [15] have described HIV risks for regular female partners of men who inject drugs, while other studies from Chennai [8,10] and Manipur [12,16] have demonstrated HIV transmission from HIV-positive men who inject drugs to their regular sexual partners, with HIV prevalence among the regular partners ranging from 16% in Chennai (in 2003) [8,12] to 45% in Manipur (in 1996/97) [12].

The Northeast Indian states of Manipur and Nagaland have been responding to the dual problems of injecting drug use and a consequent HIV epidemic for more than a decade [6]. It is estimated that 4% of adult males in Manipur and 3% in Nagaland are injecting drugs, and high levels of needle and syringe sharing have been reported [6]. According to HIV Sentinel Surveillance, HIV prevalence among PWID in these two states has ranged between 28.6% in 2008/09 and 12.9% in 2010/11 in Manipur, and between 8.4% in 2003 and 1.9% in 2007 in Nagaland [5]. A cross-sectional survey of 1,700 PWID in selected districts of Manipur and Nagaland in 2006 reported an HIV prevalence of 23% in Bishnupur district and 32% in Churachandpur district of Manipur and <2% in two districts of Nagaland [17]. Conversely, the prevalence of syphilis was lower in Manipur (5.7% in Bishnupur district, 0.9% in Churachandpur district) compared with Nagaland (7.4% in Phek, 19.5% in Wokha) [17]. HIV prevalence among women attending antenatal clinics in Manipur has ranged between 1.7% in 2004 and 0.5% in 2009, and in Nagaland between 2% in 2005 and 0.7% in 2010/11 [5]. As in the rest of India, the HIV prevention response in Manipur and Nagaland is led by the government through the National AIDS Control Organization (NACO) and the respective State AIDS Control Societies. Alongside this, Avahan (the Bill & Melinda Gates Foundation’s HIV initiative in India) funded Project ORCHID to coordinate a range of local non-government organisations to implement HIV prevention interventions in selected districts of Manipur and Nagaland over a 10-year period (2004/14) [18].

The majority of PWID in Manipur and Nagaland are male, and many have regular female sexual partners including wives, most of whom do not inject drugs [19]. As has been reported elsewhere in India [8-10], wives are often not aware of their husband’s injecting behaviour at the time of marriage, the majority remain faithful to their husbands and most do not know their husbands’ HIV status. Given the high prevalence of HIV and syphilis among men who inject drugs in Manipur and Nagaland, it is important to understand more about their sexual behaviours generally and the HIV risks for their regular female sexual partners in particular. The objectives of this study were as follows:

1. To describe the sexual behaviours of men who inject drugs in Manipur and Nagaland and
2. To describe the HIV risks for the regular female sexual partners of male PWID.

While previous studies have highlighted the problem of HIV risks for the regular sexual partners of men who inject drugs in India [8-10,12,15,16], the weight of evidence needs to be stronger so that appropriate preventive interventions can be designed, funded and implemented. Our study directly contributes to this body of evidence and provides an update of the situation in Manipur and Nagaland. Information from studies such as this one can be used to advocate for and develop effective HIV prevention programs that protect not only men who inject drugs but also their regular sexual partners.

## Methods

Data for this study were obtained from two separate cross-sectional surveys: the Integrated Behavioural and Biological Assessment (IBBA) conducted in two districts of Manipur (Churachandpur, Bishnupur) and Nagaland (Wokha, Phek) and the Behavioural Tracking Survey (BTS) also conducted in two districts of Manipur (Chandel, Ukhrul) and Nagaland (Kiphere, Zunheboto). Both surveys collected information from men who inject drugs during 2009 using an interviewer-administered questionnaire and the same sampling approach (discussed below). As the BTS questionnaire was adapted from the IBBA questionnaire, many of the questions were the same, so it was feasible to combine data pertaining to variables common to both datasets, incorporating all eight districts. The methods for both the IBBA and BTS have been described in-depth elsewhere [20,21]. The inclusion criteria for both surveys were being male aged 18 years or older and having injected drugs for non-medical purposes at least once during the last 6 months. The IBBA was a central component of the Avahan programme’s evaluation strategy [22], and the BTS was implemented by Project ORCHID to evaluate the impact of its programme in non-IBBA districts.

### Sampling

Both the IBBA and the BTS surveys used respondent-driven sampling (RDS) to recruit study participants. In brief, RDS is a sampling method based on social network theory and devised for more representative recruitment of hidden populations such as PWID [23-25]. Respondent-driven sampling uses peer networks for recruitment of participants and involves payment of purposively recruited ‘seed’ participants, who then refer other participants. All seed participants are given uniquely coded coupons to recruit three eligible participants from their personal networks. The new recruits are invited to attend a nominated RDS site, taking along their coded coupons. These new participants are in turn provided with recruitment coupons to share with their networks. This peer-to-peer participant recruitment process continues until the desired sample size is achieved. All participants are compensated for their participation and for new recruits linked to them.

For both the IBBA and BTS, a sample size of 400 per district was estimated based on an ability to detect changes in proportions of 15% at follow-up surveys from estimated baseline values of 50% (which yield the biggest sample size), an alpha level of 0.05 and a power of 90%. A design effect of 1.5 was applied to account for intra-class correlation. A total of 1,650 participants were recruited for the IBBA (Churachandpur 411, Bishnupur 410, Phek 418, Wokha 411), and a total of 1,712 participants were recruited for the BTS (Ukhrul 421, Chandel 415, Kiphere 427, Zunheboto 449). The combined dataset included a total of 3,362 PWID, which represent approximately 10% of the estimated number of PWID in the two states [26].

### Data collection

An anonymous, interviewer-administered, structured questionnaire was used to gather information regarding socio-demographics, drug use, sexual and injecting risk behaviours, knowledge of HIV and exposure to interventions. Additionally, the IBBA survey collected blood specimens that were tested for HIV and a range of STIs including syphilis. A regular partner was defined as a regular non-paid sexual partner such as a wife, spouse or girlfriend, and consistent condom use was defined as every time. Sero-prevalence of HIV infection was determined by using two test algorithms at the state laboratory (screening test: Microlisa—HIV by J.Mitra and Co. Pvt. Ltd.; confirmatory test: Genedia HIV1/2 ELISA 3.0 by Greencross Life Sciences Corp) [27].

### Statistical analysis

Descriptive data obtained using RDS is typically analysed using purpose-designed statistical software (RDSAT) to account for the complex sampling design. However, it is not possible to undertake bivariate or multivariable analysis using RDSAT, although this is possible using standard statistical software, such as Stata or R, with weights generated in RDSAT using the RDS network coding system. As our analyses are based on data combined from two RDS surveys conducted in eight districts across Manipur and Nagaland, we did not believe it would be legitimate to treat the data as one large RDS study because the RDS data collection coding systems were unique to each district. All analyses were performed using SPSS version 21 without adjustment for the complex sampling design. Consequently, the results should be viewed as if the sample were a large convenience one. The chi-square test was used to examine differences between categorical variables and the independent-sample *t* test for differences between continuous variables. Both bivariate and multivariable logistic regression analyses generated odd ratios (OR) with 95% confidence intervals (CI). We speculated that having a regular sexual partner may reduce the risk of engaging in unsafe sexual and injecting behaviours, so compared participants with regular partners to those without in relation to these behaviours, excluding those who had never had sex. We also examined the HIV risks for the regular partners of those men from the IBBA dataset who were found to be HIV positive.

Binary logistic regression modelling was used to identify factors associated with inconsistent condom use with regular female sexual partners. Variables considered for inclusion in the model were tested for co-linearity with the outcome variable, and co-linear variables such as ‘consistent condom use with casual partners’ were excluded. The variables ultimately included in the model were state, age group, literacy, marital status, shared at last injection, generally injects with needle and syringe used by another, number shared with during the last month, number of female sexual partners in the last year, frequency of sex with regular partner in the last month, length of relationship with regular partner, had casual sex in the past year, ever had an HIV test and receiving condoms from an NGO in the last year.

### Ethics

Ethical clearance for the IBBA was obtained in India through the Ethical Review Boards of the participating institutions: FHI360, the Regional Medical Research Centre (RMRC) in Dibrugarh and the National AIDS Research Institute (NARI). Ethical clearance for the BTS was obtained from the Institutional Review Board of the Emmanuel Hospital Association (EHA), New Delhi. Written informed consent was obtained from all participants prior to administration of the behavioural questionnaire and biological testing, and confidentiality was assured.

## Results

### Demographic and other background information

The total sample size was 3,362 (1,657 Manipur, 1,705 Nagaland). The mean age of participants was 27.8 years (29.6 years in Manipur, 26.2 years in Nagaland; median 26 years; range 18–57 years). Literacy was relatively high at 84.6%, although it was better in Manipur (90.9%) than in Nagaland (78.4%). Half of the sample was employed, and the other half was either unemployed or students. Sixty percent had never been married, and one third was currently married (Table 1).

**Table 1:** **Background information for men who inject drugs in Manipur and Nagaland, by state (2009)**

**Variable**	**Manipur (** ***N*** **= 1,657)**	**Nagaland (** ***N*** **= 1,705)**	**Total**
	***n*** **(%)**	***n*** **(%)**	***n*** **(%)**
District			
Churachandpur	411 (24.8)		411 (12.2)
Bishnupur	410 (24.7)		410 (12.2)
Chandel	415 (25.0)		415 (12.3)
Ukhrul	421 (25.4)		421 (12.5)
Wokha		411(24.1)	411 (12.2)
Phek		418 (12.5)	418 (12.4)
Kiphere		427 (25.0)	427 (12.7)
Zunheboto		449 (26.3)	449 (13.4)
Age (years)^a^			
<20	39 (2.4)	130 (7.6)	169 (5.0)
20–29	846 (51.1)	1,135 (66.6)	1,981 (58.9)
30–39	621 (37.5)	399 (23.4)	1,020 (30.3)
≥40	151 (9.1)	41 (2.4)	192 (5.7)
Literate^a^			
No	150 (9.1)	368 (21.6)	518 (15.4)
Yes	1,507 (90.9)	1,337 (78.4)	2,844 (84.6)
Employment status^a^			
Unemployed	602 (36.4)	764 (44.9)	1,366 (40.7)
Student	78 (4.7)	223 (13.1)	301 (9.0)
Employed	976 (58.9)	716 (42.0)	1,692 (50.4)
Marital status^a^			
Married	605 (36.5)	514 (30.2)	1,119 (33.3)
Widowed/divorced/separated	151 (9.1)	61 (3.6)	212 (6.3)
Never married	901 (54.4)	1,127 (66.2)	2,028 (60.4)

^a^ *p* < 0.001.

The mean duration of injecting was 4.9 years (median 2 years; range 0–34 years; 6.5 years in Manipur, 3.3 years in Nagaland). Heroin was the drug most commonly injected in Manipur (95.2%), while other drugs, particularly Spasmo-proxyvon (a pharmaceutical synthetic narcotic containing dextropropoxyphene), were more common in Nagaland (84.0%). Slightly less than half of the participants overall (45.6%) injected at least once daily, but this was true for two thirds in Manipur (67.5%). The practice of sharing needles and syringes was relatively common, especially in Nagaland. Overall, 16.6% of participants reported sharing at their last injection, but 30.2% did so in Nagaland, and 34.0% said they generally injected with a syringe previously used by someone else (no state differences). Overall, 27.0% had shared with at least one other during the last month, but this was true for 40.5% in Nagaland (Table 2).

**Table 2:** **HIV risk information for men who inject drugs in Manipur and Nagaland, by state (2009)**

**Variable**	**Manipur (** ***N*** **= 1,657)**	**Nagaland (** ***N*** **= 1,705)**	**Total**
	***n*** **(%)**	***n*** **(%)**	***n*** **(%)**
Injecting behaviours			
Duration of injecting (years)^a^			
≤1	285 (17.3)	471 (27.8)	756 (22.6)
2–5	636 (38.5)	943 (55.7)	1,579 (47.2)
6–10	422 (25.6)	234 (13.8)	656 (19.6)
>10	307 (18.6)	46 (2.7)	353 (10.6)
Most common drug injected^a^			
Heroin	1,578 (95.2)	273 (16.0)	1,851 (55.1)
SP and others	79 (4.8)	1,432 (84.0)	1,511 (44.9)
Injects at least once daily^a^			
No	525 (32.5)	1,174 (77.9)	1,699 (54.4)
Yes	1,090 (67.5)	333 (22.1)	1,423 (45.6)
Shared NS at last injection^a^			
No	1,598 (96.5)	1,118 (69.8)	2,716 (83.4)
Yes	58 (3.5)	484 (30.2)	542 (16.6)
Generally injects with a previously used syringe			
No	1,089 (66.0)	1,113 (66.1)	2,202 (66.0)
Yes	562 (34.0)	571 (33.9)	1,133 (34.0)
Number of people shared with during the last month^a^			
None	1,368 (86.3)	939 (59.5)	2,307 (73.0)
≥1	217 (13.7)	638 (40.5)	855 (27.0)
Sexual behaviours			
Ever had sex^a^			
No	205 (12.4)	136 (8.0)	341 (10.1)
Yes	1,452 (87.6)	1,568 (92.0)	3,020 (89.9)
No. of female partners in the last year*^a^			
None	301 (20.7)	112 (7.3)	413 (13.8)
One	758 (52.2)	588 (38.2)	1,346 (45.0)
2–5	329 (22.7)	653 (42.5)	982 (32.8)
≥6	64 (4.4)	185 (12.0)	249 (8.3)
Has a regular partner*^a^			
No	608 (41.9)	351 (22.5)	959 (31.8)
Yes	844 (58.1)	1,208 (77.5)	2,052 (68.2)
Time with regular partner^a^			
<1 year	104 (12.3)	307 (25.9)	411 (20.2)
≥1 year	739 (87.7)	880 (74.1)	1,619 (79.8)
No. of times had sex with regular partner in the last month^a^			
0	182 (21.6)	64 (5.3)	246 (12.0)
1–5	415 (49.2)	720 (59.6)	1,135 (55.3)
6–10	143 (16.9)	282 (23.3)	425 (20.7)
>10	104 (12.3)	142 (11.8)	246 (12.0)
Used condom with regular partner last time			
No	486 (58.6)	716 (59.9)	1,202 (59.4)
Yes	343 (41.4)	479 (40.1)	822 (40.6)
Consistent condom use with regular partner			
No	754 (90.6)	1,054 (88.3)	1,808 (89.3)
Yes	78 (9.4)	139 (11.7)	217 (10.7)
Had casual sex in the last year*^a^			
No	1,104 (76.0)	864 (55.8)	1,968 (65.6)
Yes	348 (24.0)	684 (44.2)	1,032 (34.4)
Used condom with casual partner last time^a^			
No	128 (36.1)	173 (24.9)	301 (28.6)
Yes	227 (63.9)	523 (75.1)	750 (71.4)
Consistent condom use with casual partners^a^			
No	280 (78.7)	472 (67.6)	752 (71.3)
Yes	76 (21.3)	226 (32.4)	302 (28.7)
Paid for sex in the last year*^a^			
No	1,258 (86.6)	1,468 (94.2)	2,726 (90.6)
Yes	194 (13.4)	90 (5.8)	284 (9.4)
Used condom with a paid partner last time^a^			
No	15 (7.7)	23 (24.7)	38 (13.2)
Yes	179 (92.3)	70 (75.3)	249 (86.8)
Consistent condom use with paid partners			
No	101 (52.1)	57 (61.3)	158 (55.1)
Yes	93 (47.9)	36 (38.7)	129 (44.9)
Ever had sex with a man^a^			
No	1,590 (96.1)	1,686 (99.0)	3,276 (97.6)
Yes	64 (3.9)	17 (1.0)	81 (2.4)
HIV and syphilis			
Feels at risk of HIV			
No	780 (49.6)	734 (51.5)	1,514 (50.6)
Yes	791 (50.4)	690 (48.5)	1,481 (49.4)
Ever had an HIV test^a^			
No	777 (47.0)	941 (58.0)	1,718 (52.4)
Yes	877 (53.0)	681 (42.0)	1,558 (47.6)
HIV positive**^a^			
No	568 (69.2)	817 (98.6)	1,385 (83.9)
Yes	253 (30.8)	12 (1.4)	265 (16.1)
Syphilis positive**^a^			
No	789 (96.1)	718 (86.6)	1,507 (91.3)
Yes	32 (3.9)	111 (13.4)	143 (8.7)
Programme exposure			
Received condoms from NGO^a^			
No	628 (37.9)	777 (45.9)	1,405 (41.8)
Yes	1,029 (62.1)	927 (54.4)	1,956 (58.2)
Received NS from NGO^a^			
No	203 (12.3)	631 (37.0)	834 (24.8)
Yes	1,454 (87.7)	1,074 (63.0)	2,528 (75.2)

*NS* = needle and syringe/s.

^a^ *p* < 0.001.

*Among those reporting ever having sex. **Those from the IBBA database only.

HIV prevalence among the IBBA participants was 16.1%, and 8.7% had reactive syphilis serology, but major state differences were observed. HIV prevalence in the two districts of Manipur was 30.8% compared with 1.4% in the two districts of Nagaland. Conversely, 13.4% in Nagaland had reactive syphilis serology, compared with 3.9% in Manipur. Participation in HIV testing was sub-optimal as 52.4% had never had an HIV test, but uptake of HIV testing in Manipur (53.0%) was better than in Nagaland (42.0%). Overall, 75.2% had received needles and syringes from an NGO, and 58.2% had received condoms, but coverage of both these services was much better in Manipur (Table 2).

### Sexual behaviours of men who inject drugs in Manipur and Nagaland

Most participants (89.9%) were sexually experienced. Among those who had ever had sex, the mean number of female sexual partners during the previous year was 2.23 (median 1; range 0–40), and more than two thirds (68.2%) had a regular female sexual partner. One tenth (9.4%) had paid for sex in the last year, and 34.4% had casual sex in the last year. Condom use with both casual and paid partners was sub-optimal: less than half (44.9%) consistently used condoms with their paid partners and only 28.7% with their casual partners. The proportion reporting having ever had sex with a man was small (2.4%). Participants from Nagaland were generally more sexually active than those from Manipur as evidenced by more sexual partners, more sex with their regular sexual partners, and greater likelihood of casual sex, while the Manipuri participants were more likely to pay for sex and to report sex with a man (Table 2).

### HIV risks for the regular female sexual partners of men who inject drugs in Manipur and Nagaland

Of the 68.2% who had a regular female sexual partner, 79.8% had been with this partner for at least 1 year. Most (88.0%) reported sex with their regular partner in the last month, on average five times (median 2; range 0–61). Condom use with regular partners was poor: 40.6% used a condom the last time they had sex with their regular partners, and only 10.7% reported consistent condom use with their regular partners (Table 2).

Many participants with regular partners (40.2%) had more than one sexual partner in the last year, 29.5% had casual sex and 6.1% paid for sex in the last year. Consistent condom use with casual sexual partners was 32.9% and with paid partners was 36.5%. Half of those with regular partners (51.0%) had never had an HIV test, and 14.3% were HIV positive (Table 3).

**Table 3:** **Factors associated with inconsistent condom use with regular partners among PWID in Manipur and Nagaland (2009)**

**Variables**	***n*** **(%)**	**Logistic regression**	
		**Unadjusted OR (95% CI)**	**Adjusted OR (95% CI)**
State			
Manipur	844 (41.1)		
Nagaland	1,208 (58.9)	0.78 (0.58, 1.05)	0.78 (0.54, 1.13)
Age group			
<30 years	1,209 (58.9)		
≥30 years	843 (41.1)	**2.56 (1.84, 3.55)**	1.19 (0.77, 1.85)
Literacy			
Illiterate	314 (15.3)		
Literate	1,738 (84.7)	**0.60 (0.38, 0.95)**	**0.58 (0.34, 0.99)**
Marital status			
Never married	923 (45.0)		
Ever married	1,127 (55.0)	**5.11 (3.66, 7.13)**	**5.23 (3.28, 8.33)**
Shared NS at last injection			
No	1,593 (79.3)		
Yes	415 (20.7)	**2.48 (1.57, 3.91)**	1.34 (0.68, 2.64)
Generally injects with NS previously used by other			
No	1,337 (65.2)		
Yes	699 (34.3)	**2.03 (1.45, 2.84)**	1.26 (0.82, 1.96)
Number of people shared with during the past month			
None	1,329 (68.5)		
≥1	610 (31.5)	**2.56 (1.75, 3.75)**	**2.71 (1.51, 4.86)**
No. of female partners in the last year			
≤1	1,213 (59.8)		
>1	816 (40.2)	**0.57 (0.43, 0.76)**	0.85 (0.52, 1.39)
No. of times had sex with regular partner in the last month			
≤4	1,204 (58.7)		
>4	848 (41.3)	**1.54 (1.26, 1.90)**	1.21 (0.95, 1.53)
Length of time with the regular partner			
<1 year	411 (20.2)		
≥1 year	1,619 (79.8)	**1.80 (1.31, 2.48)**	1.23 (0.86, 1.77)
Had casual sex in the last year			
No	1,436 (70.5)		
Yes	600 (29.5)	**0.68 (0.51, 0.91)**	1.43 (0.87, 2.35)
Consistent condom use with casual partners			
No	410 (67.1%)		
Yes	201 (32.9%)	**0.44 (0.02, 0.09)**	___
Paid for sex in the last year			
No	1,919 (93.9)		
Yes	124 (6.1)	1.56 (0.78, 3.13)	___
Consistent condom use with paid partners			
No	80 (63.5)		
Yes	46 (36.5)	0.25 (0.06, 1.07)	___
Ever had an HIV test			
No	1,029 (51.0)		
Yes	990 (49.0)	0.87 (0.65, 1.15)	**0.68 (0.49, 0.94)**
Given condoms by NGO in the last year			
No	827 (40.3)		
Yes	1,224 (59.7)	**0.63 (0.47, 0.85)**	**0.61 (0.43, 0.86)**
Given needle and syringe by NGO in the last year			
No	584 (28.5)		
Yes	1,468 (71.5)	0.74 (0.54, 1.04)	___
HIV status*			
Negative	854 (85.7)		
Positive	142 (14.3)	0.96 (0.54, 1.72)	___
Syphilis status*			
Negative	892 (89.6)		
Positive	104 (10.4)	1.43 (0.67, 3.03)	___

*Data from the IBBA database only.

*NS* = needle and syringe/s.

NB: Bolded results are significant at *p* < 0.05.

### Comparing HIV risk behaviours of the men who had regular female sexual partners with those who did not

The men with regular partners were more likely to have shared needles and syringes at their last injection (20.7% *cf* 9.1%; *p* < 0.001), to have shared in the last month (31.5% *cf* 20.4%; *p* < 0.001) and in fact had shared with more people in the last month (0.97 *cf* 0.48; *p* < 0.001) compared to those without a regular partner. One third of those with regular partners (34.3%) generally injected with needles and syringes previously used by others, but this was similar to the proportion with no regular partner (36.1%). Those with regular partners reported the same number of sexual partners over the past year as those with no regular partners (2.48 *cf* 2.09; NS) but were less likely to report casual (29.5% *cf* 44.5%; *p* < 0.001) and paid sex (6.1% *cf* 16.6%; *p* < 0.001). They were also less likely to consistently use condoms for paid sex (36.5% *cf* 51.2%; *p* = 0.013), but more likely with casual sex (32.9% *cf* 21.6%; *p* < 0.001).

### HIV risks for regular female sexual partners of HIV-positive men

Among the 265/1,650 participants (16.1%) who were found to be HIV positive in the IBBA survey, 57.5% had a regular female sexual partner. Most (89.1%) reported sex with their regular partner in the last month, on average of five times (median 3; range 0–30). Of these, only half (49.6%) reported using a condom the last time they had sex with their regular partner, and only 10.9% reported consistent condom use, which is much the same as the entire sample of men with regular partners. Even though 82.0% of the HIV-positive participants thought they were at risk of acquiring HIV, 38.5% had never previously been tested, so were presumably unaware of their HIV-positive status.

### Factors associated with inconsistent condom use with regular female sexual partners

Inconsistent condom use with regular partners was associated with being older, being illiterate, being married, unsafe injecting (at last injection, generally and sharing with others), having fewer female sexual partners, having more frequent sex and a longer relationship with the regular partner, not having casual sex, inconsistent condom use with casual partners, and not receiving condoms from an NGO (Table 3). After controlling for confounding, inconsistent condom use with regular partners was associated with being illiterate (OR 0.58; 95% CI 0.34, 0.99), being married (OR 5.23; 95% CI 3.28, 8.33), sharing needle and syringe with others in the past month (OR 2.71; 95% CI 1.51, 4.86), never having had an HIV test (OR 0.68; 95% CI 0.49, 0.94) and not receiving condoms from an NGO (OR 0.61; 95% CI 0.43, 0.86) (Table 3). Those participants who had been married were five times more likely to be inconsistent condom users with their regular partners, and those who had shared with at least one other in the last month were almost three times more likely to be inconsistent condom users. Those who were literate and those who had received condoms from an NGO were 40% less likely to be an inconsistent condom user with their regular partner, and those who had previously had an HIV test were 30% less likely.

## Discussion

The findings from this large cross-sectional dataset highlight that the majority of men who inject drugs in Manipur and Nagaland were engaging in risk behaviours, including sexual risk behaviours, that placed themselves and their regular and non-regular female sexual partners at risk of HIV infection.

Men who inject drugs in these two states were clearly sexually active. A large majority of participants (90%) had experienced sex, and most of them had been sexually active in the previous year, with 41% reporting two or more sexual partners during that time. The participants from Nagaland were more sexually active than those from Manipur, which may be due to a range of factors including younger age, less chronic opiate dependence and relatively infrequent use of heroin. In contrast to studies of sexual behaviour among PWID in other parts of India [11,14], very few reported male-to-male sexual experiences. Same-sex relationships are strongly proscribed in these traditional societies, especially in Nagaland, which may contribute to relatively fewer same-sex encounters as well as under-reporting of such behaviours. Despite the implementation of scaled interventions and consequent reductions in HIV risk behaviours [21], consistent condom use with all partner types and the uptake of HIV testing were far from optimal.

The majority of the men in this study had regular female sexual partners, half of whom were wives, and the risk of HIV transmission to these partners via unprotected sex remains. Only 41% of participants said they had used a condom the last time they had sex with their regular partner, which is the same as the proportion reported in a 2010 study among 300 men who had injected drugs from three Northeast Indian states [28]. Consistent condom use with regular partners was only 11% overall (9% in Manipur and 12% in Nagaland), which is particularly concerning in Manipur where HIV prevalence among PWID is high. In a 2007 study among men who inject drugs in Northeast Indian states, consistent condom use with regular partners was reported to be 19%, which indicates that the situation is not improving [11].

The men with regular partners were placing themselves and their partners at risk of HIV through both unsafe injecting and unsafe sexual behaviours. Those with regular partners were just as likely to be sharing needles and syringes as those without and were actually more likely to report sharing at the most recent injection and to have shared with more people. A recently published study from Northeast India also observed a positive association between unsafe injecting and unprotected sex with regular partners [13]. Some with regular partners were sexually active outside of their regular relationship, and while they reported less casual and paid sex than those with no regular partner, and condom use with casual and paid partners was better than with regular partners, it was still not optimal.

After controlling for confounding, inconsistent condom use with regular partners was associated with being married, poor literacy, sharing needles and syringes in the last month, having never had an HIV test and not receiving condoms from an NGO. Consistent condom use in the context of a regular committed relationship, especially marriage, is notoriously difficult to achieve for many people, not only PWID [29,30]. Marriages are generally based on a shared desire for intimacy and mutual trust: consequently, introducing condoms into the relationship is often unwanted and difficult to navigate (especially if the couple are wanting to have children), so a range of alternative strategies have to be made available for the wives and other regular sexual partners of men who inject drugs. The association between more consistent condom use and having ever had an HIV test could be due to the fact that some of the men who had previously been tested knew themselves to be HIV infected, but when the sub-set of HIV-positive participants are examined, there seems to be no difference between them and HIV-negative participants in terms of condom use. Alternatively, the risk reduction counselling provided at the time of HIV testing may be promoting better condom use. Also, it is probably the case that those who had contact with NGOs were both more likely to receive condoms and to be referred for HIV testing, suggesting that NGOs have a positive role to play in promoting safer sex among PWID.

A number of interventions could strengthen harm reduction programs in relation to the prevention of HIV transmission due to unprotected sex. The findings indicate that while three quarters of the participants are receiving needles and syringes from NGOs, only 58% are receiving condoms. It is probable that distribution of condoms alone is not sufficient to promote safer sexual behaviours; changes in attitudes at the individual, couple and social levels [7,8,31] and the development of negotiation skills are also required. Peer educators in harm reduction programs are not necessarily trained to facilitate such changes. It is probable that the current HIV prevention programs in these two states could be strengthened in relation to the promotion of safe sex among PWID, especially as the findings indicate that PWID who receive condoms from NGOs are more likely to be consistent condom users with their regular partners. Peer educators could be up-skilled so that they are as comfortable, conscientious and competent talking about safe sex as they are about safe injecting. It is also important that the messages are conveyed using media that will be easily understood by those with poor literacy. Evidence from a meta-analysis of the effectiveness of 33 US-based interventions that aimed to reduce the sexual risk behaviours of drug users demonstrated that targeted interventions can contribute to sexual risk reduction [32]. Additionally, the development of protocols and practices to assist men who inject drugs to disclose their HIV risks and status to their wives would be valuable, as they often find it difficult to do this alone [10].

Ensuring easy access to HIV testing for PWID is important for both them and their partners, and clearly the current level of uptake is not optimal. More than one third of the HIV-positive participants with regular partners had never had an HIV test, and those who had previously had a test may have done so while they were still HIV negative, or may not have returned for the results. Consequently, a significant proportion of the HIV-positive participants would have been unaware of their status. It was also evident that those PWID who had previously had an HIV test were more likely to use condoms consistently with their regular partner. Other studies have reported a higher proportion of consistent condom use among PWID who know they are HIV positive [29]. Identifying those men who are HIV infected and referring them for antiretroviral therapy (ART) will benefit them as well as their regular partners, as men on treatment are much less likely to transmit HIV to others [33]. While acknowledging that the concept of ‘treatment as prevention’ (TasP) is an appealing one, the effectiveness of this approach among HIV-infected PWID has not been adequately demonstrated to date [34,35].

Reaching the regular partners of men who inject drugs in order to provide HIV prevention services is challenging because contact with these women is inevitably mediated by their male partners. Not all men who inject drugs are willing to bring their regular partners to attend such services [10], and not all regular partners are interested in attending. Additionally, some HIV prevention programs are not mandated or funded to extend their services directly to the regular partners of men who inject drugs, unless they are engaging in injecting drug use or sex work. Raising awareness of HIV risks, prevention, diagnosis and treatment among regular partners of men who inject drugs is important if the HIV epidemic is to be effectively addressed. In the context of India, several resources focusing on HIV prevention among regular female sexual partners of men who inject drugs are available, and these can be used to inform the development of relevant policies and programs [28,36,37].

This study has a number of limitations that should be considered when interpreting the findings. We have combined data collected across eight districts for the IBBA and BTS surveys to construct a large convenience sample, and consequently, our analyses did not account for the complex sampling design. The limitation of this approach is that the results are essentially based on a convenience sample with restricted generalisability of the observed associations. However, the sample size was quite large, accounting for approximately 10% of the PWID population in the two states [26]. Secondly, it is highly probable that the participants’ responses to some questions regarding their injecting and sexual risk behaviours were influenced by social desirability bias, and this may have contributed to an under-estimation of HIV risks for them and their partners. No data were collected directly from regular female sexual partners themselves, so the study conclusions are based on data collected from their male partners. Finally, since these data were collected, several HIV prevention programs have endeavoured to improve coverage for the regular partners of PWID in these two states and there have been targeted campaigns to increase the uptake of HIV testing among PWID [38]. However, the impact of these subsequent interventions has not yet been evaluated, and the findings reported in this paper provide an important point of comparison for any subsequent evaluations of more recent interventions.

## Conclusion

In summary, the findings from this study involving a large number of men who inject drugs in the Northeast Indian states of Manipur and Nagaland highlight the potential risk of HIV infection for them and their regular female sexual partners. While effectively reaching the regular sexual partners of men who inject drugs is challenging, a number of strategies for improving their situation are available. These findings add to a growing body of evidence supporting the need for government and non-government agencies to strengthen the capacity of harm reduction programs to promote safer sex among PWID, improve their uptake of HIV testing and extend HIV prevention services to their regular sexual partners, especially in areas of the country where HIV prevalence among PWID is high.
